# Tumour-infiltrating neutrophils counteract anti-VEGF therapy in metastatic colorectal cancer

**DOI:** 10.1038/s41416-018-0198-3

**Published:** 2018-10-31

**Authors:** Lars Mortimer Schiffmann, Melanie Fritsch, Florian Gebauer, Saskia Diana Günther, Neil Richard Stair, Jens Michael Seeger, Fabinshy Thangarajah, Georg Dieplinger, Marc Bludau, Hakan Alakus, Heike Göbel, Alexander Quaas, Thomas Zander, Frank Hilberg, Christiane Josephine Bruns, Hamid Kashkar, Oliver Coutelle

**Affiliations:** 1 Cologne Excellence Cluster on Cellular Stress Responses in Aging-Associated Diseases (CECAD), Center for Molecular Medicine Cologne (CMMC) and Institute for Medical Microbiology, Immunology and Hygiene, University of Cologne, CECAD Research Center, Joseph-Stelzmann-Str. 26, 50931 Cologne, Germany; 2 Department of General, Visceral and Cancer Surgery, University of Cologne, Kerpener Str. 62, 50937 Cologne, Germany; 3 Center for Integrated Oncology (CIO) Cologne Bonn, Gastrointestinal Cancer Group Cologne (GCGC), Kerpener Str. 62, 50924 Cologne, Germany; 4 Department of Gynaecology and Obstetrics, University of Cologne, Kerpener Str. 34, 50931 Cologne, Germany; 5 Institute for Pathology, University of Cologne, Kerpener Str. 62, 50937 Cologne, Germany; 6 Department I of Internal Medicine, University of Cologne, Kerpener Str. 62, 50924 Cologne, Germany; 7 Boehringer Ingelheim RCV, Doktor-Boehringer-Gasse 5-11, 1120 Vienna, Austria

**Keywords:** Colorectal cancer, Predictive markers, Tumour angiogenesis

## Abstract

**Background:**

Immune infiltration is implicated in the development of acquired resistance to anti-angiogenic cancer therapy. We therefore investigated the correlation between neutrophil infiltration in metastasis of colorectal cancer (CRC) patients and survival after treatment with bevacizumab. Our study identifies CD177+ tumour neutrophil infiltration as an adverse prognostic factor for bevacizumab treatment. We further demonstrate that a novel anti-VEGF/anti-Ang2 compound (BI-880) can overcome resistance to VEGF inhibition in experimental tumour models.

**Methods:**

A total of 85 metastatic CRC patients were stratified into cohorts that had either received chemotherapy alone (*n* = 39) or combined with bevacizumab (*n* = 46). Tumour CD177+ neutrophil infiltration was correlated to clinical outcome. The impact of neutrophil infiltration on anti-VEGF or anti-VEGF/anti-Ang2 therapy was studied in both xenograft and syngeneic tumour models by immunohistochemistry.

**Results:**

The survival of bevacizumab-treated CRC patients in the presence of CD177+ infiltrates was significantly reduced compared to patients harbouring CD177− metastases. BI-880 treatment reduced the development of hypoxia associated with bevacizumab treatment and improved vascular normalisation in xenografts. Furthermore, neutrophil depletion or BI-880 treatment restored treatment sensitivity in a syngeneic tumour model of anti-VEGF resistance.

**Conclusions:**

Our findings implicate CD177 as a biomarker for bevacizumab and suggest VEGF/Ang2 inhibition as a strategy to overcome neutrophil associated resistance to anti-angiogenic treatment.

## Introduction

Anti-angiogenic treatment with bevacizumab, an antibody that targets vascular endothelial growth factor (VEGF), has become an integral part of solid cancer therapies.^1^ However, many patients develop treatment resistance^2^ and no clinical or biological factors have been identified that clearly predict which patients respond or develop resistance to bevacizumab (bev). Several mechanisms of resistance to anti-angiogenic treatments have been identified.^3^ Amongst them, the compensatory activation of the angiopoietin/Tie-2 axis has been proposed as one of the main drivers of resistance.^4^ We have previously shown that elevated angiopoietin-2 (Ang2) levels counteract the therapeutic effects of bev in colorectal cancer patients^5^ and that the combined inhibition of both VEGF and Ang2 improved tumour growth control and vascular normalisation.^6^ These findings confirmed Ang2 as a promising anti-angiogenic target in conjunction with VEGF-targeting therapy.

More recent evidence suggests that the recruitment of innate immune cells into the tumour can foster a pro-angiogenic environment, representing another mode of resistance to anti-angiogenic treatments.^7–9^ Strikingly, anti-VEGF treatment appeared to trigger the recruitment of myeloid cells into the tumour that conferred resistance to anti-angiogenic treatment especially in colorectal cancer.^10^ This treatment resistance could be overcome, however, when immune cell recruitment was disrupted.^11^ Besides its direct effect on tumour vessels, the Ang2/Tie-2 axis has also been shown to interfere with immune cell infiltration in tumours. In particular, Ang2 inhibition reduced the number of intra-tumoural Tie-2+monocytes, thereby increasing the efficacy of anti-angiogenic drugs^12^ leading to efficient blockade of angiogenesis, tumour growth and metastasis.^13^ While the above-mentioned observations refer to experimental cancer models, a number of investigations in colorectal cancer patients concerning the prognostic relevance of immune cell infiltration remained contradictory.^14–16^ Notably, none of these studies stratified for patients who received anti-angiogenic therapy.

To address this issue, we compared immune cell infiltration in colorectal cancer patients who subsequently received bevacizumab with patients who did not. We stained CD177 as a neutrophil surface molecule that is stably expressed in neutrophils independent of gender, age or activation state, making it a reliable marker for tumour-infiltrating neutrophils in the circulation, e.g., from the primary tumour site to metastatic lesions.^17^ Here we identified CD177-positive neutrophil infiltration as a predictor of adverse clinical response to bevacizumab treatment. Furthermore, we show in a mouse xenograft model of colorectal cancer that the novel bi-specific VEGF/Ang2 blocking nanobody BI-880 diminished neutrophil infiltration associated with anti-VEGF treatment and efficiently reduced tumour growth and hypoxia. Finally, we demonstrate that neutrophil depletion or BI-880 treatment can overcome resistance to VEGF inhibition in a syngeneic lung cancer model. Our data explore the combined inhibition of VEGF/Ang2 as a novel and valuable strategy to disrupt the adverse effects of reactive myeloid recruitment following VEGF blockade and demonstrate its potential for vascular normalisation and tumour growth control.

## Materials and methods

### Mouse xenograft model

The human colorectal cancer cell line LS174T was purchased from ATCC and cultured as previously described.^6,18^ Animals were obtained from Janvier. To generate xenografts, 8-week-old female BALBc nude mice were subcutaneously injected with 3 × 10^6^ LS174T cells into the flank. After palpable tumour development of at least 50 mm^3^, mice were randomised into treatment groups and exposed to either vehicle, bevacizumab (Roche, 5 mg/kg, subcutaneously (s.c.), twice weekly) or BI-880 (Boehringer Ingelheim, 4 mg/kg or 16 mg/kg, both intraperitoneally (i.p.), twice weekly), respectively. Tumour volume was measured every other day and calculated using the formula *v* = 3.14/6 × length × width^2^.

### Syngeneic subcutaneous tumour model

Murine Lewis lung cell carcinoma cells (LLC) were purchased from ATCC and maintained in Dulbecco's modified Eagle's medium supplemented with 10% foetal calf serum and 1% penicillin/streptomycin. The 8-week-old C57BL/6 mice were subcutaneously injected with 1.5 × 10^6^ LLC cells into the right flank. After palpable tumour development of at least 50 mm^3^, mice were randomised into treatment groups and exposed to either vehicle, B20 (B20-4.1.1 creative biolabs, 5 mg/kg s.c., twice weekly), 16 mg/kg BI-880 plus 5 mg/kg B20 (both i.p., twice weekly) or B20 in combination with an anti-Ly6G antibody (1 mg/ml, 3×/week, InVivo plus, clone 1A8), respectively. Anti-Ly6G treatment started 1 day before tumour cell injection (compare Fig. 5a). Tumour volume was measured every other day and calculated using the formula *v* = 3.14/6 × length × width^2^.

### Ethics statement

All animal experiments were performed in accordance with the German animal protection law as approved by local government authorities. Animals were housed in the animal care facility of the University of Cologne under standard pathogen-free conditions with a 12 h light/dark schedule and provided with food and water ad libitum.

### Immunofluorescence stainings

Xenograft tumour tissue was cryo-sectioned (20 µm) and processed as previously described.^6^ Endothelial cells were stained with a monoclonal Armenian-hamster anti-mouse CD31 antibody (1:200, Abcam) or a fluorescein isothiocyanate (FITC)-labelled anti-CD31 antibody (1:200, clone 390, Biolegend), and apoptotic cells were detected with a rabbit anti-cleaved caspase-3 (Asp175) antibody (1:200, Cell Signaling). Pericytes were detected either by a rabbit anti-NG2 Chondroitin Sulphate Proteoglycan antibody (1:100, Millipore) or a rat anti-PDGF-R antibody (1:100, CD140b, eBioscience). Vascular endothelial (VE) cadherin was stained with a rat anti-CD144 (VE-cadherin) antibody (BD Pharmingen). The following secondary antibodies derived from goat were used to label the respective primary antibodies with fluorescent dyes: Alexa Fluor 647 anti-hamster, Alexa Fluor 594 anti-rat, Alexa Fluor 594 anti-rabbit and Alexa Fluor 488 anti-hamster (1:500, Thermo Fisher Scientific). Vascular leakage was quantified by analysing intravenously injected FITC-dextran (molecular weight 2000 kDa, Sigma), pimonidazole adducts were detected with a FITC-labelled anti-pimonidazole antibody to visualise hypoxia; both procedures have been previously described in detail^6,19^; nuclei were stained with 4,6-diamidin-2-phenylindol (DAPI).

### Immunofluorescence microscopy and analysis

Vessel density was expressed as the number of CD31+ vessels/visual field counted on representative 100× images of tumour sections. Vascular leakage was calculated as dextran-positive area/CD31-positive area of 100× images using ImageJ software (http://imagej.nih.gov/ij). Pericyte coverage was obtained from 600× images (NG2) or 100× images (PDGFRβ) and quantified using the ImageJ colocalisation plugin as previously described elsewhere.^20^ VE-Cadherin expression was analysed on 600× images stained for VE-Cadherin and CD31 as previously described.^6^ Hypoxia was quantified as the pimonidazole positive area using 40× images of tumour sections. Tumour cell death was calculated as the sum of apoptotic (identified as cleaved caspase-3 positive) and necrotic (Pimonidazole−DAPI negative) tissue area, necrosis was confirmed via haematoxylin and eosin stainings. Neutrophils were counted (LS174T) or electronically quantified using ImageJ (LLC) on representative 100× images. Imaging was performed on a motorised inverted microscope (Olympus IX81 equipped with Cell^R Imaging Software, Tokyo, Japan) using different objectives as indicated.

### Human colorectal cancer samples

Samples were collected at the University Hospital of Cologne with written informed consent from patients with colorectal cancer concurring with the Declaration of Helsinki.

### Tissue microarray construction, CD177 staining and analysis

The tissue microarray (TMA) was constructed consisting of 558 tumour samples from patients with colorectal cancer who had been treated between 1999 and 2014 at the Department of General, Visceral and Cancer Surgery, University of Cologne. Stage IV patients were stratified depending on whether they subsequently had received adjuvant conventional chemotherapy (ctx) alone or in conjunction with bevacizumab after the respective tissue sample was collected; from these stage IV patient samples from either lymph node metastasis from the primary tumour resection or distant organ metastases from lung or liver resulting in a total of 85 samples (*n* = 39 ctx only group; *n* = 46 bev-containing group) which were included in this study. TMA construction was performed as previously described.^21^ In brief, tissue cylinders with a diameter of 1.2 mm each were punched from selected tumour tissue blocks using a self-constructed semi-automated precision instrument and embedded in empty recipient paraffin blocks. The 4 μm sections of the resulting TMA blocks were transferred to an adhesive-coated slide system (Instrumedics Inc., Hackensack, NJ) for immunohistochemistry.

Immunohistochemistry to stain CD177 as a marker for tumour-infiltrating neutrophils was performed on TMA slides using the BOND MAX from Leica (Wetzlar, Germany) according to the manufacturer's protocol. We used the monoclonal mouse anti-CD177 antibody (clone 4C4; M01; Abgent) at a dilution of 1:200 as a primary antibody.

### Strategy of evaluation

The CD177 scoring was performed in a blinded fashion by a high-level trained pathologist with the following values: 0 = negative: up to 9% positive neutrophils, 1 = 10–24% positive neutrophils and 2 = >25% positive neutrophils. Score 0 was considered CD177 negative. Scores 1 and 2 were considered CD177 positive.

### Statistical analysis

All results are expressed as mean ± standard error of the mean (s.e.m.). Statistical differences between experimental groups were calculated using the unpaired Student’s *t*-test or one-way analysis of variance (for tumour growth curve). Survival curves were plotted using the Kaplan–Meier method and statistically analysed by log-rank test. All tests were two-sided. N.S. indicated not significant (*p* > 0.05); *p < 0.05 was considered as statistically significant and **p < 0.01 as highly significant. Symbols above bars show statistical significance vs. the control group. Symbols above horizontal lines indicate significance between the connected groups.

## Results

**Fig. 1:**
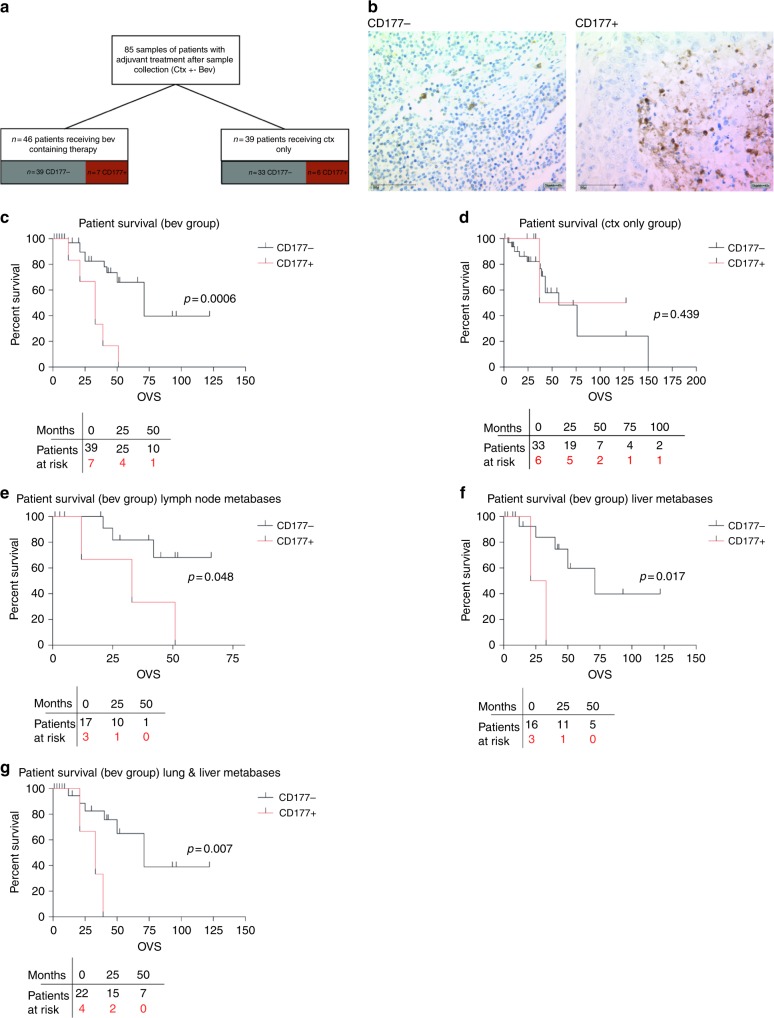
Survival of different subgroups of colorectal cancer (CRC) patients. **a** Schematic depicting criteria for inclusion in the retrospective analysis and **b** representative images of CD177− or CD177+ scored metastases. **c** Kaplan–Meier curves of patients who received bev in their history stratified for CD177 score on TMAs of CRC metastasis (lymph node and organ metastases) or **d** who received a bev-free treatment regimen in their history stratified for CD177 score on TMAs of metastasis (lymph node and organ metastases). **e**–**g** Survival curves of samples derived from **e** lymph node metastases only or **f** liver metastases or **g** lung and liver metastases. The *p* values were determined by log-rank testing

To address whether the presence of tumour-infiltrating myeloid cells was associated with the progression of colorectal cancer patients who received anti-angiogenic therapy, consecutive patients with colorectal cancer treated in our centre were identified from an internal database and a TMA was constructed consisting of 558 tumour samples. Stage IV patients were stratified depending on whether they subsequently had received adjuvant conventional chemotherapy (ctx group) alone or in conjunction with bevacizumab (bev group). For the latter, only patients were included who were bev naive before the respective resection; a total of 85 samples (*n* = 39 ctx only group; *n* = 46 bev-containing group) were included (Fig. 1a). Table 1 depicts the basic characteristics of the studied cohorts; differences between the ctx and the bev groups were only apparent regarding number of treatments and the frequency of liver resections.

**Table 1:** Basic characteristics of studied patients

	Ctx	Bev	*P*
Metastatic patients	39	46	
Sex			
Female	12	14	1
Male	27	32	
Age at diagnosis	59.3	58.7	0.82
Stage (UICC)			
I–III	0	0	1
IV	39	46	
Distribution of metastatic sites			
Nodal	23	20	0.051
Liver	9	22	
Lung	7	4	
Mean follow-up (months)	88.6	87.4	0.88
Average no. of treatment lines	1.79	2.54	0.008
Patients with available primary tumour material	25	17	
Localisation of the primary tumour			
Rectum	11	4	0.391
Rectosigmoid, sigma, colon descendens	8	7	
Colon ascendens	6	6	
Initial T stage			
pT1	0	0	0.706
pT2	1	0	
pT3	14	10	
pT4	10	7	
Initial N stage			
pN0	2	2	0.753
pN1	10	5	
pN2	13	10	
Liver resection			
Yes	16	30	0.033
No	21	15	
N.A.	2	1	

*Ctx* chemotherapy only, *Bev* chemotherapy plus bevacizumab, *UICC* union internationale contre le cancer

### Metastatic CD177 status predicts outcome upon bev treatment

We first analysed tumour neutrophil infiltrates by immunostaining of CD177+ cells on TMAs derived from colorectal metastasis samples (Fig. 1b), including samples from lymph node (*n* = 23 ctx only group; *n* = 20 bev group), lung or liver (*n* = 16 ctx only group; *n* = 26 bev group) metastases. Strikingly, we found that neutrophil infiltration was associated with poor outcome of patients who were treated with bev (Fig. 1c) Overall survival (OVS) in the bev-treated cohort was found to be significantly reduced in patients with CD177+ infiltrates compared with CD177− samples. Median OVS in patients with a negative score was 71 months, which dropped to 33 months (46.5%; 95% confidence interval 46.4–95.6 vs. 19.4–46.6; *p* = 0.0006) in patients with CD177+ metastasis (Fig. 1c). This was not the case in patients who received chemotherapy without bev (Fig. 1d). Interestingly, we observed a significant difference in prognosis depending on the CD177 status in lymph node metastases collected during the primary surgical resection (Fig. 1e). This also holds true for later stages in lung/liver metastases (Fig. 1f, g).

### The novel bi-specific VEGF/Ang2 neutralising nanobody BI-880 effectively blocks tumour growth and vascularity

**Fig. 2:**
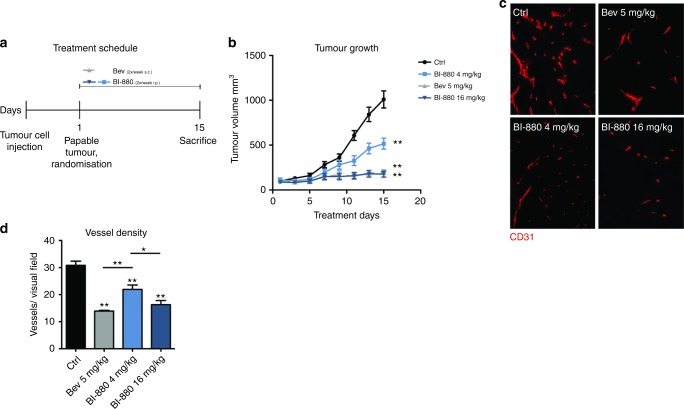
Effect on LS174T tumour growth and vascularisation. **a** Schematic depicting animal experimental procedure. **b** Growth curves of subcutaneous LS174T xenograft tumours. Tumour-bearing mice were treated with either vehicle, bev or BI-880 (4 mg/kg or 16 mg/kg) for 14 days. **c** Representative 100× images depicting vascularisation of LS174T tumour sections stained with CD31 (red). **d** Quantification of vessel density. *p < 0.05, **p < 0.01

To gain mechanistic insights as to how anti-angiogenic therapy impacts neutrophil infiltration in colorectal cancer, we employed a subcutaneous xenograft model of human colorectal cancer cells (LS174T) in BALB/c nude mice. Tumour-bearing mice were treated with either vehicle, bev (5 mg/kg) or the bi-specific VEGF/Ang2 neutralising compound BI-880 in two different doses (4 mg/kg (BI-880^4^) or 16 mg/kg (BI-880^16^)); treatment and schedule is depicted in Fig. 2a. BI-880^4^ and BI-880^16^ both inhibited tumour growth significantly compared to controls. BI-880^4^ showed an intermediate reduction, whereas BI-880^16^ resulted in effective tumour control comparable to bev treatment (Fig. 2b). Corresponding to the decrease in tumour growth, the respective treatments were associated with a reduction in tumour microvascularisation (Fig. 2c, d).

### BI-880 avoids hypoxia-triggered neutrophil infiltration which occurs upon bev

**Fig. 3:**
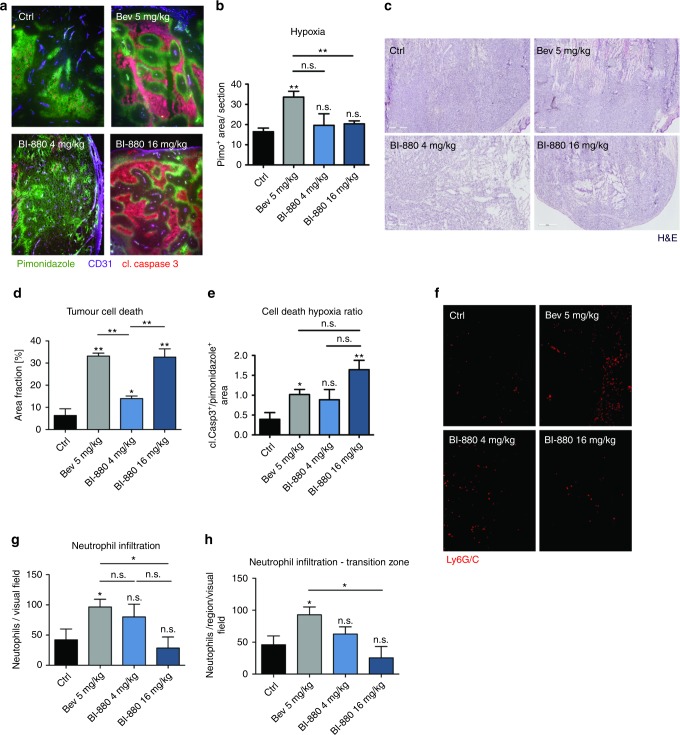
Impact on hypoxia, cell death and neutrophil invasion in colorectal cancer xenografts. **a** Images (40×) of hypoxic tumour area (pimonidazole adducts, green), tumour cell death (cl. caspase-3, red) and adjacent blood vessels (CD31, purple). **b** Quantification of overall tumour hypoxia **c** haematoxylin and eosin (H&E) staining of tumour sections depicting necrotic tumour area and **d** quantification of tumour cell death and **e** cell death/hypoxia ratio. **f** Images of xenograft-infiltrating neutrophils (Ly6G/C red, 100×) and **g** quantification thereof; **h** quantification of tumour-infiltrating neutrophils in the transition zone from hypoxia to necrotic tissue. *p < 0.05, **p < 0.01, n.s. not significant

To discover changes in intra-tumoural hypoxia and cell death caused by vessel regression upon treatment with bev, BI-880^4^ or BI-880^16^ tumour sections were stained with a pimonidazole adduct detecting antibody (to identify hypoxic tissue) and an antibody against cleaved caspase-3 (to detect apoptotic cell death). Surprisingly, BI-880^4^ and BI-880^16^ did not result in increased hypoxia compared with untreated controls (Fig. 3a, b) despite a significant reduction in vessel density (Fig. 2c, d). In contrast, bev therapy increased the hypoxic tumour fraction as a consequence of reduced vessel density (Fig. 3a, b). Similarly, cell death levels were increased in both bev- and BI-880^16^-treated tumours (Fig. 3c, d) but by comparison, reduced hypoxia in the BI-880^16^ tumours resulted in a higher death/hypoxia ratio (Fig. 3e). This difference could be crucial as hypoxia can drive the recruitment of myeloid-derived suppressor cells (MDSCs), like neutrophils, that may adversely influence the clinical outcome.^22^ Exploring this hypothesis, we analysed the abundance of Ly6G/C+ myeloid-derived cells in the xenografts and found a significant increase in neutrophil count in bev- but not BI-880^16^-treated tumours (Fig. 3f, g). Indeed, neutrophils accumulated nearly entirely in the transition zone between hypoxia and necrosis, suggesting that hypoxia could be driving the recruitment of these myeloid cells (Fig. 3h).

### BI-880 augments vascular normalisation

**Fig. 4:**
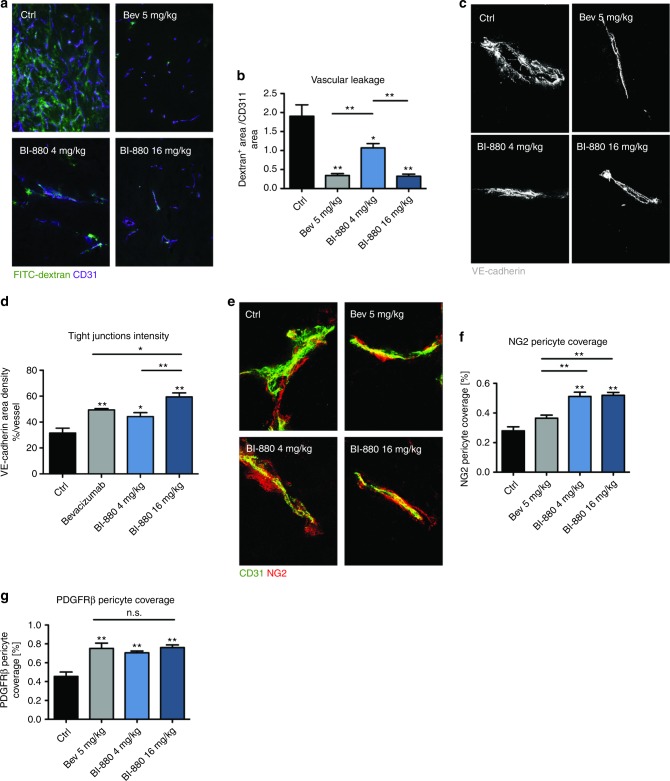
Treatment-induced vessel normalisation. **a** Images of FITC-dextran (green) perfused tumour blood vessels (purple) and **b** quantification of vascular leakage. **c** High-magnification images (600×) of VE-Cadherin (grey) stained tumour vessel endothelium and **d** quantification of tight-junction integrity. **e** High-magnification images (600×) of tumour blood vessels stained for CD31 (green, EC) and NG2 (red, pericytes) and **f** quantification of NG2 pericyte coverage and **g** quantification of PDGFRβ pericyte coverage. *p < 0.05, **p < 0.01, n.s. not significant

Both VEGF and Ang2 blockade induce complex tumour vessel alterations that have been summarised in the vascular normalisation theory.^23^ We therefore examined changes in the vascular barrier as a function of vascular normalisation in bev-, BI-880-^4^ or BI-88^16^-treated tumour vessels. For that purpose, tumour-bearing mice were i.v. injected with FITC-labelled dextran and extravasation into the tumour tissue was monitored as a measure of vascular permeability. Vascular leakage was significantly reduced in all treatment groups compared with controls. Leakage in BI-880^4^ tumours was intermediate between bev and BI-880^16^ and the control group (Fig. 4a, b). Consistent with the functional improvement of endothelial barrier function, vascular tight junctions appeared to be more regular as indicated by the linear VE-Cadherin pattern in the respective treatment groups (Fig. 4c, d). Increased pericyte coverage is another recognised parameter of vascular normalisation. Indeed, pericyte coverage as detected by two different markers (NG2 and PDGFR-β) was significantly increased upon treatment with bev, BI-880^4^ or BI-880^16^ (Fig. 4e–g).

### BI-880 overcomes anti-VEGF resistance

**Fig. 5:**
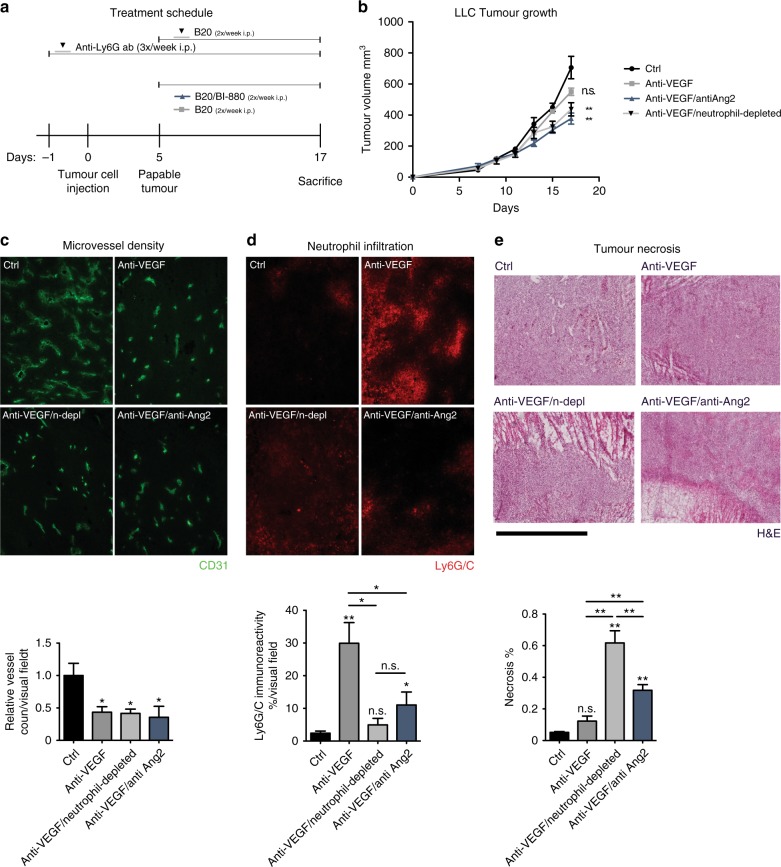
BI-880 overcomes anti-VEGF resistance in LLC tumours. **a** Schematic depicting treatment schedule. **b** Tumour growth of syngeneic LLC tumours subjected to vehicle, anti-VEGF (B20), anti-VEGF/anti-Ang2 (B20/BI-880) or anti-VEGF/anti-neutrophil (B20/anti-Ly6G antibody) treatment. **c** Representative 100× images depicting vascularisation of LLC tumour sections stained with FITC-CD31 (green) and quantification of vessel density. **d** Images of xenograft-infiltrating neutrophils (Ly6G/C red, 100×) and quantification thereof. **e** H&E staining of tumour sections and quantification of tumour necrosis (scale bar 2000 µm); *p < 0.05. **p < 0.01, n.s. not significant

To investigate if BI-880 treatment could overcome MDSC-mediated resistance to VEGF inhibition as suggested by our findings in patients and experimental xenografts, we next employed the syngeneic LLC tumour model that is known to be resistant to anti-VEGF treatment. LLC cells were injected subcutaneously into wild-type C57BL/6 mice which were subsequently treated either with vehicle or an antibody against murine VEGF (B20, Genentech) or BI-880^16^ plus B20 (BI-880 effectively binds to both human and murine Ang2 and human but not murine VEGF) or B20 combined with depletion of tumour-infiltrating neutrophils using an anti-Ly6G antibody (Fig. 5a). The tumour growth rates were unaffected by anti-VEGF treatment (B20) alone consistent with the reported anti-VEGF resistance of the LLC tumour model. Remarkably, after neutrophil depletion or addition of BI-880^16^ growth inhibition in response to anti-VEGF treatment was restored (Fig. 5b). Corresponding to our observations in the colorectal cancer patients, anti-VEGF treatment in the LLC tumour model also induced significant neutrophil recruitment (Fig. 5d). Microvessel density was similarly reduced in all three treatment groups (Fig. 5c). Although VEGF inhibition alone failed to induce significant tumour cell death in LLC cancers, significant tumour cell death was induced in response to VEGF inhibition following neutrophil depletion or BI-880 treatment (Fig. 5d).

In summary, we identified CD177 as a potential predictive biomarker for the response to bev-containing anti-cancer therapy in colorectal cancer patients and characterise a potential causal chain linking hypoxia to neutrophil recruitment upon bev treatment. Importantly, these adverse effects of VEGF inhibition may be avoided by combining both VEGF and Ang2 inhibition using the novel bi-specific drug BI-880.

## Discussion

The combination of bevacizumab and cytotoxic chemotherapy has demonstrated a survival benefit in the first- and second-line treatment of metastatic CRC. Therefore, reliable biomarkers that predict the response or resistance to bevacizumab are of great clinical interest. Here we report that the absence of CD177-positive neutrophils in colorectal cancer metastasis identifies patients with a significant survival advantage in response to bev therapy. Although the study cohort was relatively small, a clear advantage of our study is the analyses of different metastatic compartments (lymph node, organ metastases). Minor differences concerning clinical characteristics between the ctx and the bev cohorts were only observed for the number of treatment lines and frequency of liver resections, indicating a more intense treatment history for the bev cohort.

Median OVS in patients lacking CD177-positive neutrophil infiltration was 71 months vs. 33 months (46.5%; 0.0003) in patients with CD177-positive metastasis. The CD177-positive neutrophil score was predictive of survival, regardless of whether this count was conducted early on lymph node biopsies at the time of primary tumour resection or later during tumour progression in lung or liver metastases. Although lymph node metastasis represents an important prognostic factor in patients with CRC, sentinel lymph node biopsy in colorectal cancer is unreliable due to skipping lymph node metastasis, in which distant nodes may be positive but those closer to the tumour are negative. Therefore, other factors like extracapsular invasion at the N1 site have been used to predict distant regional metastasis at the N2 site.^24^ Given its prognostic value, it is tempting to speculate that CD177+ neutrophil infiltration in regional lymph nodes may also be a sensitive indicator for distant metastasis and help to identify patients who could benefit from anti-angiogenic therapy.

Neutrophils physiologically migrate to sites of injury to contribute to tissue homeostasis by recruiting additional cells of the immune system to re-vascularise and repair tissue damage.^25^ In malignancy, neutrophils are instructed by the primary tumour to migrate towards sites of metastasis in order to prepare the metastatic niche.^26^ Intra-tumuoral hypoxia is a strong stimulus for neutrophil recruitment and we found elevated hypoxia levels in bev-treated xenograft tumours with an accompanying increase in neutrophil abundance after bev treatment but not in BI-880^16^-treated tumours. These differences are entirely consistent with improved vascular normalisation resulting from dual Ang2/VEGF inhibition, which we have detailed previously^6^. Importantly, here we showed that the observed accumulation of Ly6G/C+ myeloid cells after bev treatment could be prevented by dual inhibition of VEGF and Ang2 with BI-880. Xenograft tumours treated with BI-880^16^ showed reduced intra-tumoural hypoxia and increased relative cell death compared with bev treatment despite similar reductions in vessel density and leakiness, consistent with previous findings.^6,27^ A recent experimental study in glioblastoma also showed synergistic effects for the combined inhibition of VEGF and Ang2 on tumour growth and myeloid cell recruitment.^28^ In contrast, a phase I trial for AMG 386 (trebananib)—a dual inhibitor of both Ang1 and Ang2—reported negative results in patients when combined with bev or in patients who had received bev treatment prior to the study.^29^ This may not be entirely surprising given that Ang1 and Ang2 have largely antagonistic activities, although angiopoietin signalling is complex and context dependent.^30^ A correlation between bev response and microvessel density has recently been reported for both ovarian and colorectal cancer patients where higher CD31 counts were associated with significantly better survival.^31^ Whether the higher vessel count is protective for bev-induced hypoxia and subsequent neutrophil recruitment can only be speculated.

CD177 belongs to the Ly-6 gene superfamily supporting our assumption that CD177+ neutrophils in bev-treated patients are comparable to Ly6G/C+ immune cells in xenografts^32^; this is also underlined by the fact that depletion of Ly6G+ neutrophils by an antibody could restore efficacy of anti-VEGF therapy which alone did not suffice to block tumour growth in a syngeneic mouse model. The same data also lend credibility to our conclusion that inhibition of the Ang2-/Tie-2 signalling axis can overcome myeloid compartment-driven resistance to anti-VEGF therapy.

Myeloid-derived tumour-infiltrating cells have been shown to secrete a broad spectrum of cytokines and chemokines that orchestrate pro-tumourigenic, pro-angiogenic processes during tumour progression promoting invasion and metastasis. Several studies dealt with inflammatory cells and their impact on prognosis in colorectal cancer. For example, increased neutrophil/lymphocyte ratios (NLR) in peripheral blood predicted overall survival in advanced colorectal cancer.^33^ However, these studies did not stratify for anti-angiogenic treatment. This and the fact that circulating immune cells in the blood were analysed may limit the comparability with our data. Here we investigated tumour-infiltrating neutrophils in the respective metastatic tissues. Our data suggest that it is important to discriminate between these different compartments (primary site, nodal and organ metastasis, circulation). Another recent study compared 141 patients receiving ctx plus bev and 148 patients receiving ctx alone. They found that only patients who have a low NLR benefited from bev.^34^ This is in line with our data, when considering that circulating neutrophils at least in part are representative for metastasis-infiltrating neutrophils.

In conclusion, our finding that CD177+ myeloid cells found in metastasis of colorectal cancer patients predict poor outcome in response to bev treatment could offer a new diagnostic tool to identify patients who are unlikely to benefit from bev-containing therapy or whose prognosis might be adversely affected by bev. Our findings warrant further analysis of tumour-infiltrating neutrophils as a biomarker for the development of resistance during bevacizumab treatment and to assess the value of combining Ang2/VEGF inhibition to overcome bev resistance with second-generation drugs like BI-880.

## Availability of data and material:

Datasets generated and analysed for this work are not publicly available and but are accessible upon request.
